# Radiation Resistance of Silicon Carbide Schottky Diode Detectors in D-T Fusion Neutron Detection

**DOI:** 10.1038/s41598-017-13715-3

**Published:** 2017-10-17

**Authors:** Linyue Liu, Ao Liu, Song Bai, Ling Lv, Peng Jin, Xiaoping Ouyang

**Affiliations:** 1 School of Nuclear Science and Technology, Xi’an Jiaotong University, No. 28, Xianning West Road, Xi’an, 710049 China; 2 State Key Laboratory of Intense Pulsed Radiation Simulation and Effect, Northwest Institute of Nuclear Technology, Xi’an, 710024 China; 3 Nanjing Electronic Devices Institute, Building 03, No.8 Xingwen Road, Nanjing, 210016 China; 4 School of Advanced Materials and Nanotechnology, Xidian University, Xi’an, 710071 China; 5 Shaanxi Engineering Research Center for Pulse-Neutron Source and its Application, Xijing University, Xi’an, 710123 China

## Abstract

Silicon carbide (SiC) is a wide band-gap semiconductor material with many excellent properties, showing great potential in fusion neutron detection. The radiation resistance of 4H-SiC Schottky diode detectors was studied experimentally by carefully analyzing the detectors’ properties before and after deuterium-tritium fusion neutron irradiation with the total fluence of 1.31 × 10^14^ n/cm^2^ and 7.29 × 10^14^ n/cm^2^ at room temperature. Significant degradation has been observed after neutron irradiation: reverse current increased greatly, over three to thirty fold; Schottky junction was broken down; significant lattice damage was observed at low temperature photoluminescence measurements; the peaks of alpha particle response spectra shifted to lower channels and became wider; the charge collection efficiency (CCE) decreased by about 7.0% and 22.5% at 300 V with neutron irradiation fluence of 1.31 × 10^14^ n/cm^2^ and 7.29 × 10^14^ n/cm^2^, respectively. Although the degradation exists, the SiC detectors successfully survive intense neutron radiation and show better radiation resistance than silicon detectors.

## Introduction

Since many giant scientific fusion devices, such as ITER^1–3^, EAST^4,5^, NIF^6–9^, etc. came into use in several countries in the world, the diagnostic of the neutron field in nuclear fusion plasmas has become an interesting and challenging subject. Owing to the high neutron fluence and extreme temperature in fusion devices, some of currently used semiconductor detectors based on silicon and germanium materials cannot satisfy the demands of neutron detection very well^10,11^. The germanium detectors need to operate in low temperature. The radiation resistance of silicon detectors is not ideal: significant radiation damage has been observed once the irradiation fluence reaching to 1 × 10^12^ n/cm^2^, and they are expected to be difficult to operate above neutron fluence of 1 × 10^14^ n/cm^2^ ^12,13^. New radiation detectors based on new materials have been developed. Diamond is a good detection medium, with an ultra-high neutron radiation resistance and stable properties at varying temperatures, but the applications of diamond detectors are badly restricted by the tiny dimension and high cost of high-quality diamond materials^14–19^. With the development of semiconductor technologies, silicon carbide (SiC) has been found to be an ideal material for radiation detection with better radiation resistance in intense radiation field and better stability at high temperature than silicon and germanium^20–27^. In addition, the mature of SiC preparation technology has made it possible to fabricate large high-quality SiC detectors: the largest commercial SiC wafer up to 6 inch in diameter and high-quality epitaxial film over 100 μm in thickness has been successfully fabricated.

The radiation resistance is a key parameter for SiC detectors. In this paper, the radiation resistance of SiC detectors with Schottky diode structure is discussed. The detectors were irradiated at room temperature by deuterium-tritium fusion neutrons with energy of 14 MeV and total neutron fluence of 1.31 × 10^14^ n/cm^2^ and 7.29 × 10^14^ n/cm^2^ (Table 1). Then their parameters, including the curves of forward I-V, reverse I-V and C-V, the photoluminescence, the alpha particle spectra and the charge collection efficiency (CCE) were investigated. The SiC detectors survived after fast neutron irradiation with fluence over 10^14^ n/cm^2^ showing better resistance than silicon detector.

**Table 1:** 4H-SiC detectors in neutron radiation (En = 14 MeV).

Detector Number	Neutron fluence
Detector 1#	0 (pre-irradiated)
Detector 2#	1.31 × 10^14^ n/cm^2^
Detector 3#	7.29 × 10^14^ n/cm^2^

## Experimental

### Detector fabrication

**Figure 1:**
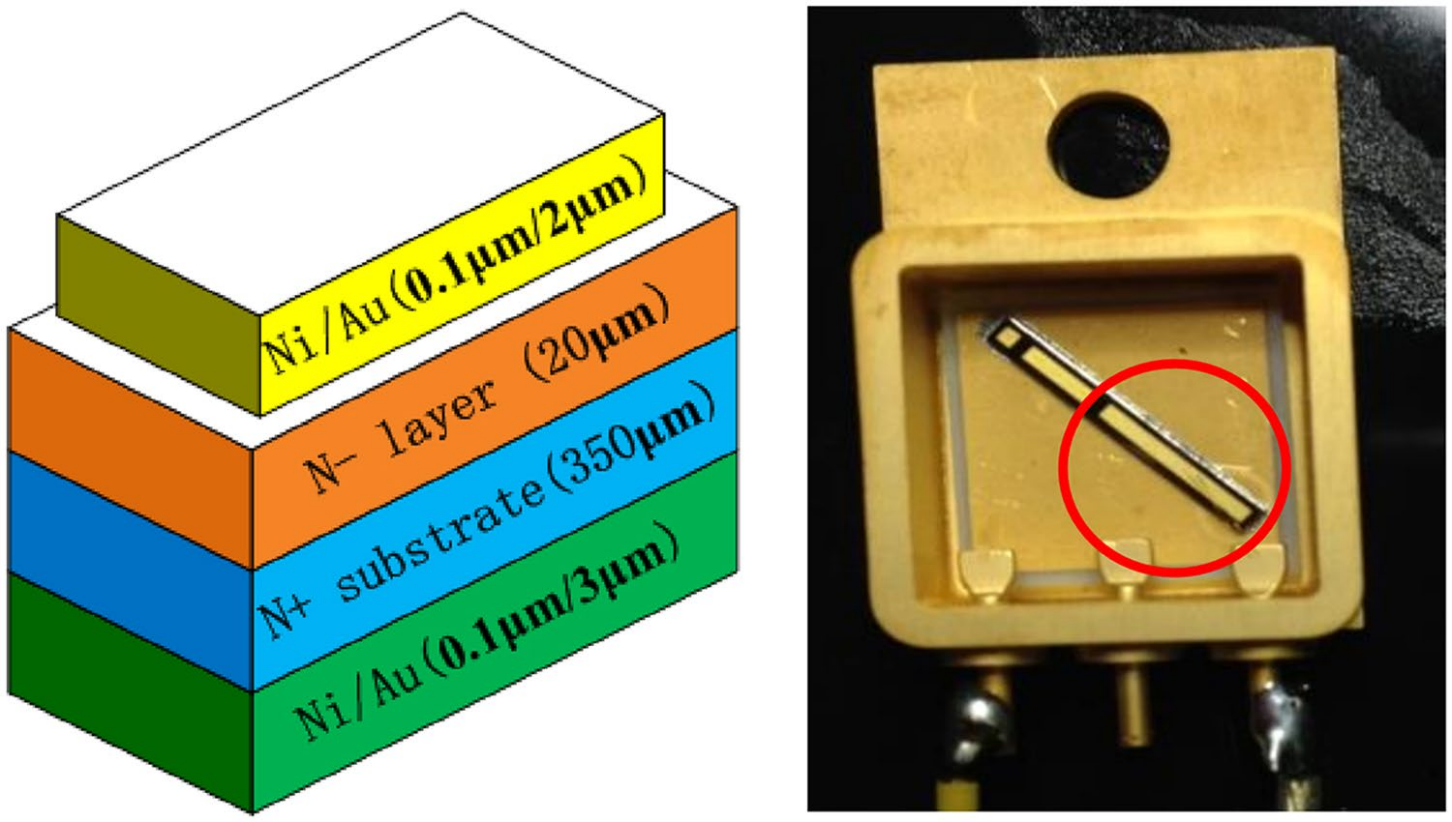
(**a**) Schematic diagram of a 4H-SiC Schottky diode which includes four layers: the Ni/Au layer in green is the ohmic back electrode, the N+ substrate in blue is the commercial 4H-SiC N+ conducting substrate wafer, the N- layer in orange is the sensitive volume of the SiC detector composited by high-quality lightly doped epitaxial 4H-SiC material grown by CVD technology and the Ni/Au layer in yellow is the front Schottky electrode. (**b**) Photograph of a 4H-SiC Schottky diode detector packaged in a ceramic shell, which is marked by a red circle and with a sensitive volume of 1mm × 5 mm × 20 μm.

Three 4H-SiC detectors (#1, #2 and #3) were fabricated with high-quality lightly doped epitaxial 4H-SiC layers grown by chemical vapor deposition (CVD) on commercial 4H-SiC N+ conducting substrate wafers (Φ 4 in. × 350 μm, target nitrogen doping concentration of 10^19^ cm^−3^). The epitaxial thickness is 20 μm and the target nitrogen doping concentration is 1 × 10^14^ cm^−3^. The front Schottky electrodes were made with Ni, 100 nm in thickness, prepared by thermal vacuum evaporation, and were coated with 2-μm-thick Au. The back ohmic contact electrodes were formed with Ni/Au (100 nm/3μm-thick). A set of multi-floating rings were made around the front contact to protect it from the damage of high voltage. All the three detectors were of Schottky structure, and have a sensitive volume of 1mm × 5 mm × 20 μm and dead layer of Ni/Au (100 nm/2 μm). The SiC diode chips were packaged in ceramic shells and were bonded with Au wires to the electrodes. Figure 1 is schematic diagram of the 4H-SiC detectors.

### Neutron radiation

The irradiation was performed at the K600 Neutron Generator in China Institute Atomic Energy (CIAE) in Beijing, China, which can provide a constant fast neutron beam generated by the deuterium-tritium fusion, with an average energy of 14 MeV and a neutron fluence rate of 4–12 × 10^9^ n/cm^2^s. Two SiC chips (Detector #2 and #3, before being packaged) were irradiated by the fast-neutron with fluence of 1.31 × 10^14^ n/cm^2^ and 7.29 × 10^14^ n/cm^2^, respectively. The radiation temperature was at 283 K.

## Results and Discussion

### I-V and C-V characteristics

**Figure 2:**
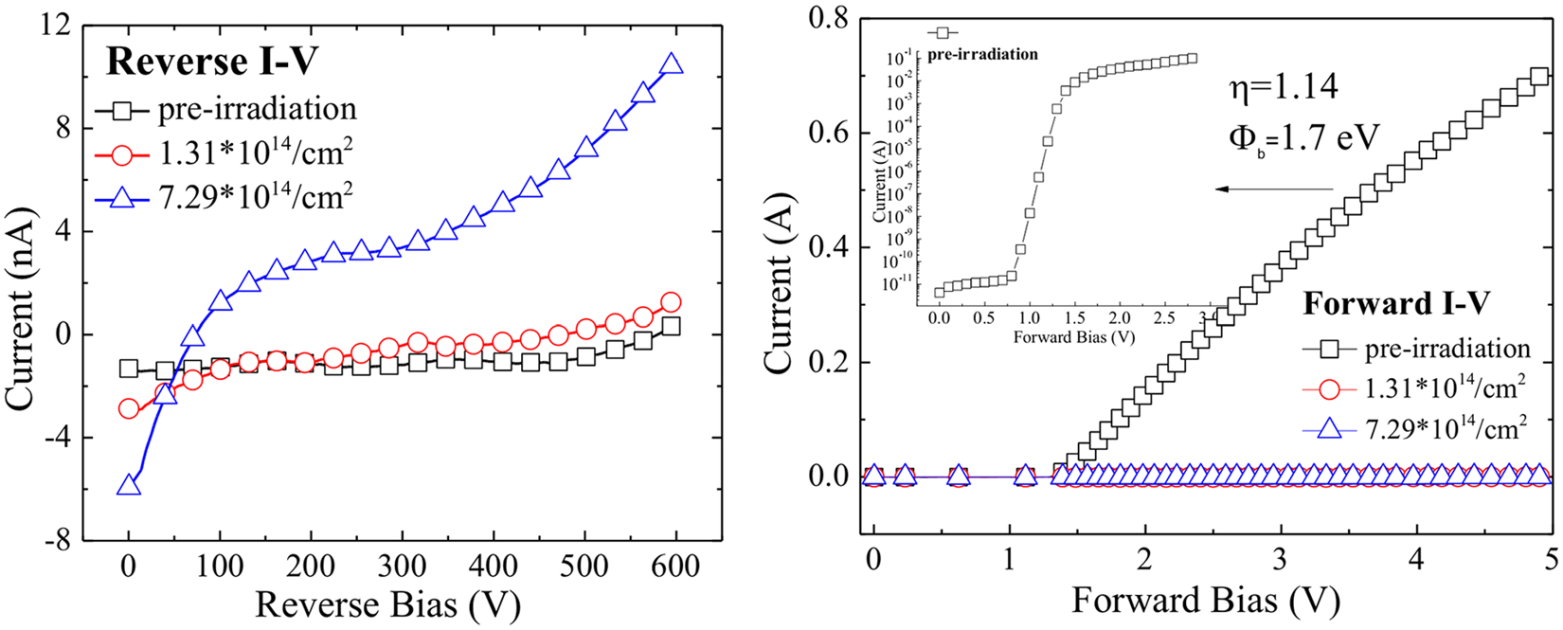
Electric parameter measurement results of the SiC diodes before (black open blocks) and after neutron irradiation (red open circles with neutron radiation fluence of 1.31 × 10^14^ cm^−2^ and blue up-triangles with neutron radiation fluence of 7.29 × 10^14^ cm^−2^): (**a**) Reverse current vs. reverse bias voltages in the range of 0 to 600 V; (**b**) Forward I-V curves for the three SiC diodes, the inset being the forward current of the diode before neutron irradiation in index vertical coordinates.

The front and reverse I-V curves were measured with IWATSU CS-3200C Curve Tracer, and the results are shown in Fig. 2. The detectors were applied with reverse bias voltages in the range of 10 V to 600 V, thus the dark current of the detectors could be expressed by the reverse current. It is found that the dark current (reverse current) of the two detectors being irradiated increased significantly with the increase of neutron fluence. At the low fluence, it increased by three times or more; at the high fluence, it increased even more greatly by more than thirty times.

The forward I-V curve of the un-irradiated diode chip shows the rectification character, but the two chips being irradiated lost that character and the Schottky junction was broken down. The forward I-V characteristics of Schottky barrier diodes can be described by the Bethe’s Thermionic emission theory, in which the effective Richardson’s constant is 146 A cm^−2^ K^−2^ for 4H-SiC^28–32^. According to the forward I-V characteristics and the Bethe equation, the ideality factor was calculated to be 1.14, which indicates the current is dominated by thermionic current. The Schottky barrier height Ф_b_ for the Ni/4H-SiC contact was calculated to be 1.7 eV.

**Figure 3:**
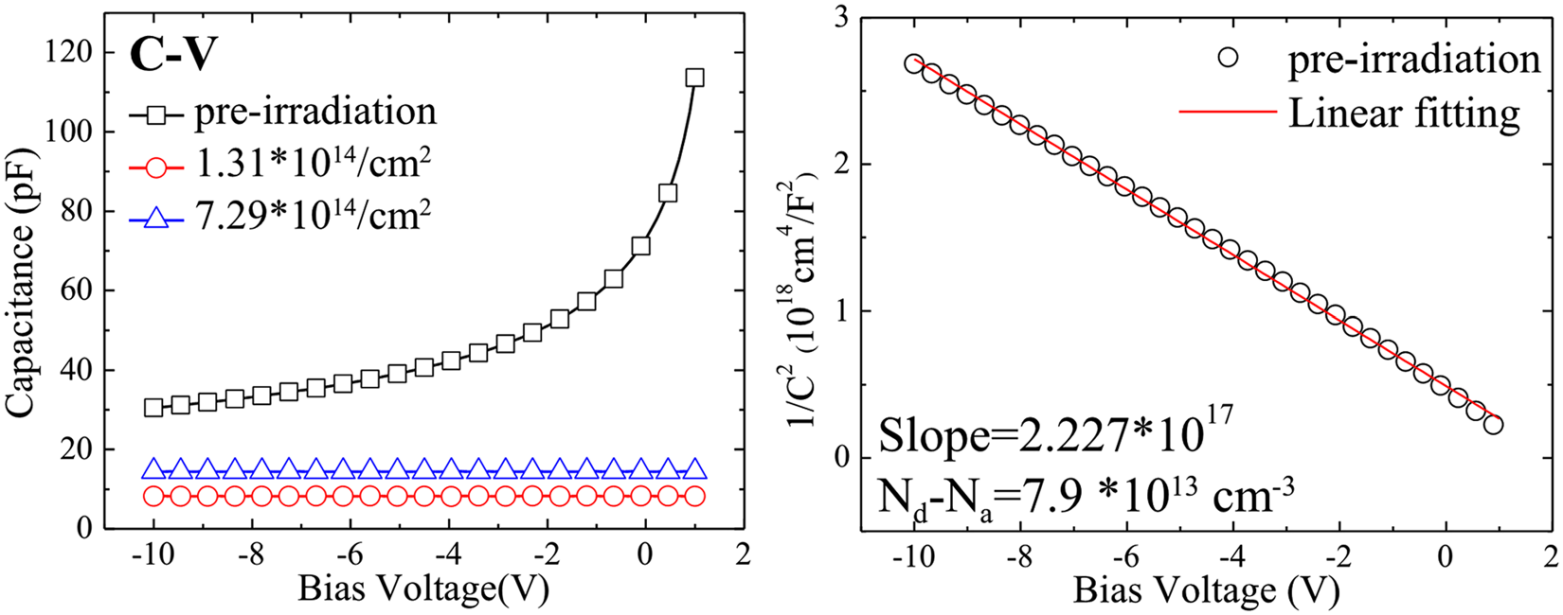
(**a**) Capacitance vs. reverse bias voltages in the range of −8V to 1 V before (black open blocks) and after neutron irradiation (red open circles with neutron radiation fluence of 1.31 × 10^14^ cm^−2^ and blue up-triangles with neutron radiation fluence of 7.29 × 10^14^ cm^−2^); (**b**) The 1/C^2^ vs. reverse bias voltages and its linear fitting before neutron irradiation.

Figure 3a shows the C-V curve acquired by Agilent B1500A Semiconductor Parameter Analyzer. Figure 3b shows the curve of 1/C^2^ vs. V of the detector #1 (un-irradiated), from which the net doping concentration of 4H-SiC epitaxial layer and the built-in voltage of the Schottky diode can be acquired. From Fig. 3, we find the two samples (#2 and #3) being irradiated lost their C-V characters, and the effective doping concentration (N_eff_) of the 4H-SiC epitaxial layer was 7.9 × 10^13^ cm^−3^ and the built-in V_bi_ potential of the Schottky contact was 1.7 V. The Schottky barrier height can be expressed as

1 $${{\rm{\Phi }}}_{b}(C-V)={V}_{bi}+{V}_{n}={V}_{bi}+kT/q\times ln\frac{{N}_{c}}{{N}_{eff}},$$

where *N*_c_ is the effective density of the states in the conduction band of 4H-SiC, here is taken as 1.7 × 10^19^ cm^−3^. The barrier height thus was calculated 2.0 eV. The difference between the barrier heights from the I-V and C-V curves are due to the following factors: barrier height obtained from the forward I-V curve was calculated with the current which flows through the Schottky barrier over the entire area where the metal electrode covers, while the one derived from the C-V curve was calculated with the average capacitance related to the whole detector. Besides, the ideality factor we got is deviated from 1, which exposes the spatial inhomogeneity of the surface barrier height.

### Photoluminescence

**Figure 4:**
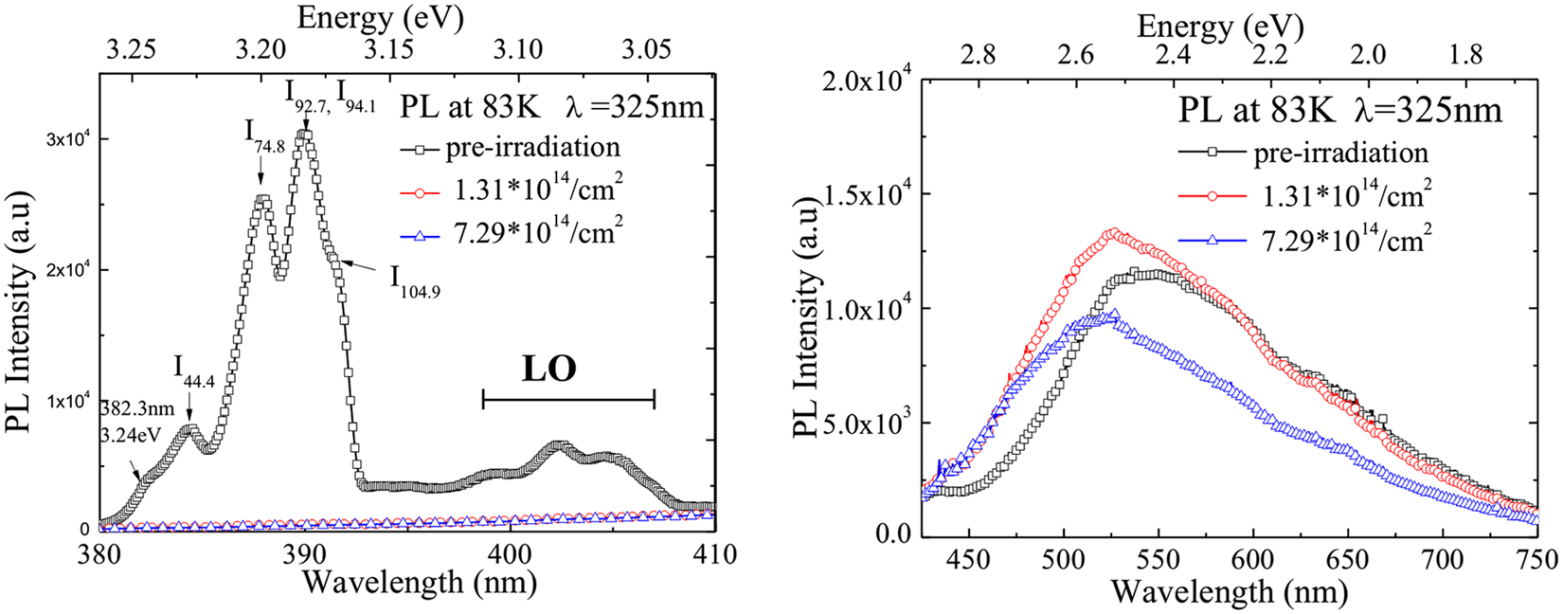
Low temperature PL spectra of 4H-SiC layer before (black open blocks) and after neutron irradiation (red open circles with neutron radiation fluence of 1.31 × 10^14^ cm^−2^ and blue open up-triangles with neutron radiation fluence of 7.29 × 10^14^ cm^−2^): (**a**) the near band edge emission intensity changed remarkably after irradiation; (**b**) variation of broad vibronic band ranging from 2.12 eV to 2.23 eV before and after irradiation.

Photoluminescence (PL) experiments were performed to detect defects in the epitaxial material of the SiC detectors. Low temperature photoluminescence (LTPL) spectra were acquired in the spectral region between 380 nm and 800 nm at temperature of 83 K–203 K. A He-Cd laser with a wavelength of 325 nm was used as the excitation light source. Figure 4 shows the integrated pulsed PL spectra of the 20-μm-thick lightly doped epitaxial 4H-SiC layer taken at 83 K, with a nitrogen concentration of about 7.9 × 10^13^ cm^−3^.

As indicated in Fig. 4a, the distribution of the PL intensity of the 4H-SiC layer changes remarkably after neutron irradiation. The PL spectrum of the detector #1 is dominated by the near-band-gap nitrogen bound exciton lines (3.15–3.24 eV) and their associated phonon replicas (LO), but for the detector #2 and #3 being irradiated, the luminescence is completely quenched. This might be attributed to the severe lattice damage induced by neutron radiation.

The PL spectra in lower energy are dominated by a broad PL peak, covering green to yellow-green spectral range, peaking at about 2.12 ~ 2.23 eV, and in the spectra of all the samples, the intense and broad vibronic bands can be observed. By reference to the reported broad PL band by Sridhara *et al*.^33^, Gao et al^34^. and Sakai *et al*.^35^, we found this broad PL band might be composed by the peaks at 2.10 eV, 2.35 eV and 2.80 eV. The peaks at 2.35 eV and 2.80 eV might be due to the donor-to-acceptor (DAP) transition from the nitrogen donor (0.1 eV below the conduction band) to the deeper and shallower boron acceptors (0.7 eV and 0.3 eV above the valence band)^36^. The peak at 2.10 eV might be due to the carbon vacancy, which is a candidate for the electron transition, or other unidentified defect level^35^. The average luminance wavelength shifts with neutron fluence, the higher the neutron fluence is, the shorter the average wavelength would be, indicating some non-radiative defects have been produced by neutron irradiation.

**Figure 5:**
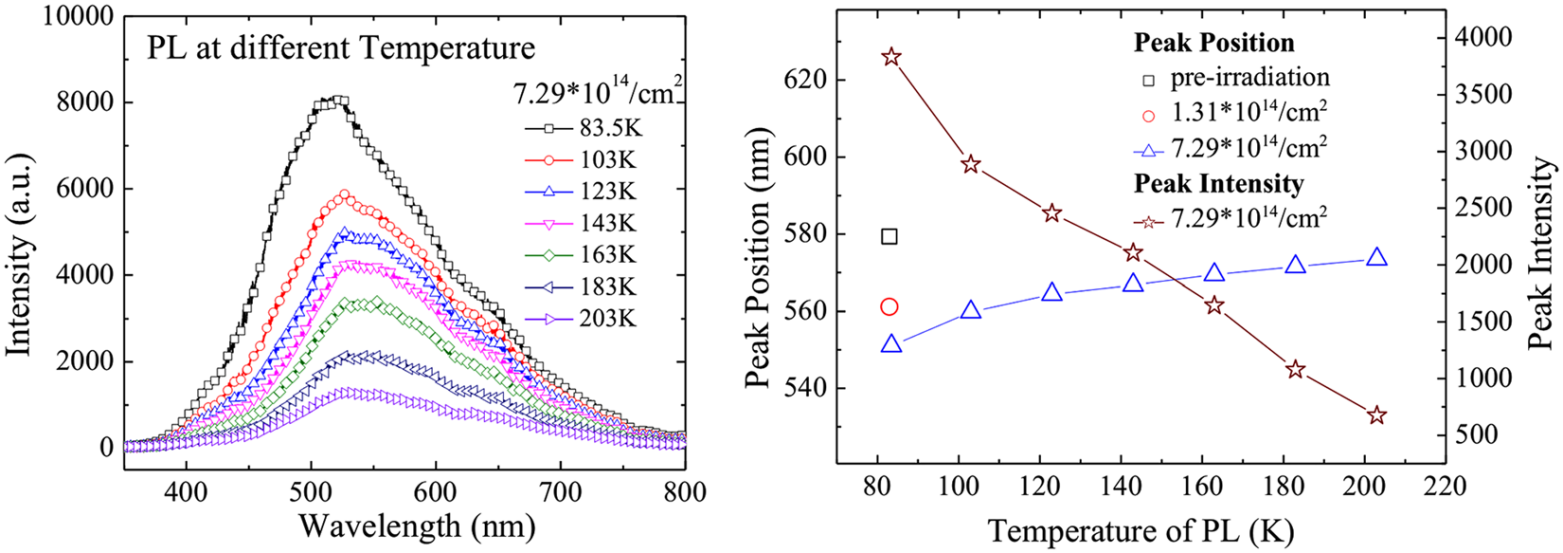
(**a**) PL of the 4H-SiC layer at different temperatures ranging from 84 K to 203 K after neutron irradiation with fluence of 7.29 × 10^14^ cm^−2^; (**b**) the variation of the peak intensity of the PL spectra at different temperatures before (black open blocks) and after neutron irradiation (blue open circles with neutron radiation fluence of 1.31 × 10^14^ cm^−2^ and blue open up-triangles with neutron radiation fluence of 7.29 × 10^14^ cm^−2^) and the position of the PL peaks after irradiated by neutrons (brown open stars) with fluence of 7.29 × 10^14^ cm^−2^ at different temperatures.

Figure 5a shows the PL of the samples measured at temperature ranging from 84 K to 203 K. Broadband green luminescence is observed in the figure. As the temperature increases, the intensity of the luminescence decreases and the luminescence band becomes broad. This possibly derives from the lattice vibration and lattice scattering. (Fig. 5b) The broadband green luminescence might be due to both the vacancies of carbon and its extended point defects, which would lead to the consistent existence of green luminescence and the variation of the intensity and wavelength at different temperatures.

### Alpha particle spectra

**Figure 6:**
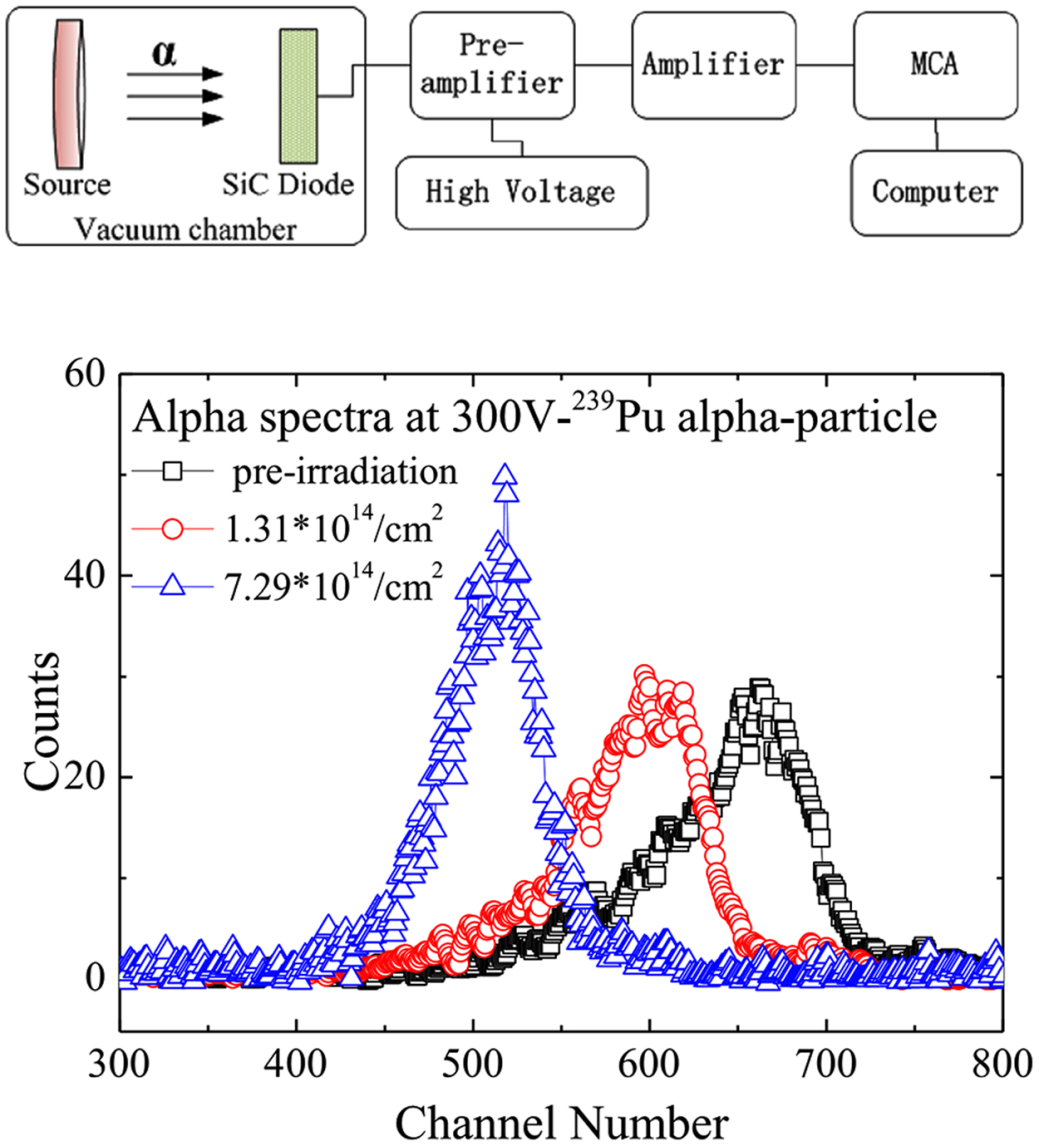
(**a**) Experimental setup for detection of the alpha particles including electronic devices such as a Preamplifier-Ortec 142B, an Amplifier Ortec 672, a High Voltage Supply- Stanford PS350, a MCA(Ortec MCA) and a laptop computer(Lenovo T420i). (**b**) response spectra to ^239^Pu alpha particles for the three SiC detectors before (black open blocks) and after neutron irradiation at a reverse bias voltage of 300 V (blue open circles with neutron radiation fluence of 1.31 × 10^14^ cm^−2^ and blue open up-triangles with neutron radiation fluence of 7.29 × 10^14^ cm^−2^) acquired at room temperature.

The detectors were tested under the irradiation of alpha particles from ^239^Pu alpha source (Φ10 mm, E_α_ = 5.157 MeV [73.3%]), 5.144 MeV[16.1%], 5.105 MeV[11.5%], 20000 Bq), provided by Northwest Institute of Nuclear Technology in Xi’an, China. The alpha source and the detectors were enclosed in a vacuum chamber, about 80 mm away from each other. The reverse bias was provided by a PS350 high voltage supply (Stanford research system Inc.) through the Ortec 142B preamplifier in the range of 0 to 300 V. Standard electronic devices, including an Ortec 142B preamplifier (with gain of 20 mV/MeV), an Ortec672 amplifier (with shaping time of 1μs and gain of 50 times) and an Ortec multi-channel analyzer (MCA), were used to record the signals. A laptop was used to obtain the pulse height spectra. The experimental setup is shown in Fig. 6(a).

According to the calculation with SRIM2003 Code^37^, the dead layer of the SiC detectors, comprised of Ni/Au (100 nm/2 μm), can absorb about 1.00 MeV of the incident alpha particles, leaving about 4.16 MeV kinetic energy penetrating into the active layer. Because the projected range of those remnant alpha particles, about 12.2 μm, is smaller than the sensitive thickness of the SiC detector (20 μm), all the remnant energy of the alpha particles would be deposited in the active layer.

The counts of the alpha particles as a function of channel number are shown in Fig. 6(b). The alpha-particle peaks can be clearly observed. The peak centroid of alpha particles is at Channel 665 for detector 1#, Channel 619 for detector 2# and Channel 516 for detector 3#. The higher neutron fluence is, the lower the peak centroid would be. The width of alpha peaks decreases with the increase of the incident neutron fluence. Fitting the peaks with Gaussian function, we got the FWHMs of the three alpha peaks, which are 391 keV for detector 1#, 384 keV for detector 2# and 270 keV for detector 3#. Excluding the influence of electronic noise (10 keV), static broadening (6.0 keV) and energy straggling of dead layer (180 keV)^27^, we got the inherent FWHM of 347 keV for detector 1#, 334 keV for detector 2# and 201 keV for detector 3#.

### Charge Collection Efficiency

**Figure 7:**
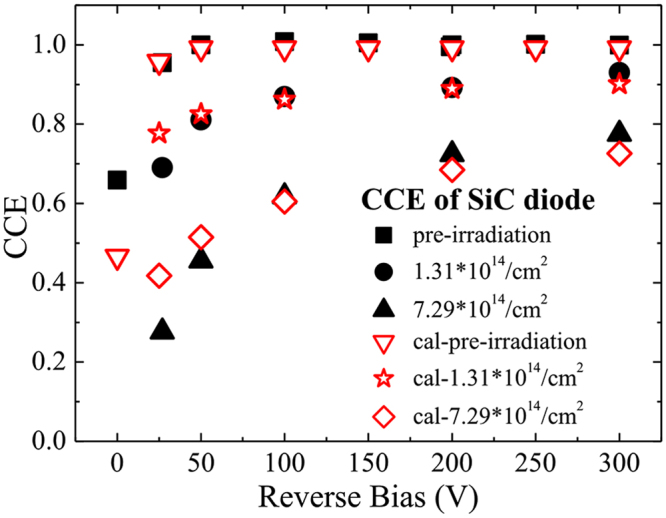
CCEs of the three SiC Schottky diodes derived from alpha particle detection experiment before (black solid blocks) and after neutron irradiation (black solid circles with neutron radiation fluence of 1.31 × 10^14^ cm^−2^ and black solid up-triangles with neutron radiation fluence of 7.29 × 10^14^ cm^−2^) and from theoretical calculations (red open down-triangles before irradiation, red open stars with neutron fluence of 1.31 × 10^14^ cm^−2^and red open diamonds with neutron fluence of 7.29 × 10^14^ cm^−2^).

Charge collection efficiency (CCE) is defined as the ratio between the numbers of the electric charges collected by the detectors (Q_c_) and the total number of electric charges of all the excited carriers (Q_g_)^28,32^. Q_g_ is dependent upon the energy of the alpha particles deposited in the detectors’ sensitive volume, about 4.16 MeV. Because all the electronic devices in our alpha detection experiments were kept working stably, the Q_g_ could be determined by the peak centroid of the detector 1# at the reverse bias voltages which could make the detector fully depleted, at Channel 665, and Q_c_ could be determined by the peak centroid of alpha peaks. Figure 7 gives the CCE of the three detectors at different bias voltages with different neutron radiation fluence.

As shown in Fig. 7, the CCE decreases with the increase of the neutron fluence, which is consistent with early researches of other scientists^28,32^. The 4H-SiC detectors couldn’t work without power supply after neutron irradiation. After the irradiation, the Schottky barrier of the detector was broken down, the 4H-SiC detector would not be depleted even if it is applied with reverse bias voltages within 300 V. The detectors are still alive after neutron irradiation but need high reverse bias voltages to avoid steep decrease of signal intensity. The CCEs of the SiC detectors decrease about 7.0% and 22.5% at 300 V when the neutron fluence reaches to 1.31 × 10^14^ n/cm^2^ and 7.29 × 10^14^ n/cm^2^, respectively. But the CCE of a silicon detector decreased much more, over 75%, at lower fast-neutron irradiation fluence of 9.98 × 10^12^ n/cm^2^ ^38^. It can be concluded that the radiation resistance of SiC detector is much better than silicon detector.

Theoretically, the CCE of the un-irradiated detector is expressed as^32^

2 $$CCE=\frac{1}{{E}_{ion}}{\int }_{0}^{d}(\frac{dE}{dx})dx+\frac{1}{{E}_{ion}}{\int }_{d}^{R}[(\frac{dE}{dx})\times \exp \{-\frac{(x-d)}{{L}_{d}}\}]dx$$

3 $$d=\sqrt{\frac{2\varepsilon {\varepsilon }_{r}({\varphi }_{b}+V)}{q{N}_{eff}}}$$

where *E*_ion_ is the energy of the incident alpha particles, 5157 MeV; *d* is the depletion width at a given bias; *dE*/*dx* is the rate of loss of energy of the implanted alpha particles as they penetrate the 4H-SiC epilayer; *R* is the projected range of the incident particles with an energy of *E*_ion_, 16 μm; *L*_d_ is the diffusion length of the minority carriers, 6.8 μm; *ε* and *ε*_*r*_ are dielectric and relative dielectric constant of the dead layer, 9.7 and 8.85 × 10^−14^ F/cm, respectively; q is 1.6 × 10^−19^ C; *N*_eff_ is 7.9 × 10^13^ cm^−3^.

The CCEs of the SiC detectors being irradiated are expressed as^28^

4 $$CCE=\frac{1}{{E}_{ion}}{\int }_{0}^{R}(\frac{dE}{dx})\frac{\frac{q}{T}\{{\lambda }_{p}[1-\exp (-\frac{x}{{\lambda }_{p}})]+{\lambda }_{n}[1-\exp (-\frac{(T-x)}{{\lambda }_{n}})]\}}{q}dx$$

where *E* is the electric field, *E* = *V/d*; *T* is the thickness of the 4H-SiC epitaxial layer, T = 20 μm; λ_p_ and λ_n_ are the mean free path of the holes and electrons, respectively, ${\lambda }_{p}={\mu }_{p}{\tau }_{p}E,\,{\lambda }_{n}={\mu }_{n}{\tau }_{n}E$, *μ* is the carrier mobility*; τ* is the carrier lifetime;. μ_n_τ_n_ and μ_h_τ_h_ are 2.2 × 10^−8^ cm^2^/V and 1.7 × 10^−8^ cm^2^/V with the low fluence of 1.31 × 10^14^cm^−2^, 1.5 × 10^−8^ cm^2^/V and 1.1 × 10^−8^ cm^2^/V with the high fluence of 7.29 × 10^14^cm^−2^. Compared with the value of μτ (over 1000 × 10^−8^ cm^2^/V) for the holes of the detector not being irradiated^28^, it decreases significantly after neutron irradiation. The decrease of μτ can be atrributed to the neutron irradiation defects, which act as trapping centers for carriers, reducing the probability of the carrier transportation through the SiC material.

The calculation result of the CCEs is plotted in Fig. 7 with open dots. They are well consistent with the experimental results acquired in the alpha particle detection.

## Conclusions

We compared the properties and performance of 4H-SiC Schottky diode detectors before and after the irradiation of deuterium-tritium fusion neutrons with total fluence of 1.31 × 10^14^ n/cm^2^ and 7.29 × 10^14^ n/cm^2^ at room temperature. We found that the 4H-SiC Schottky diode detectors being irradiated survived the intense neutron radiation, and were still effective and could be used in radiation detection even though the detector performance was degraded by the increase of dark current, reduction of CCE, decrease of μτ and movement of alpha peaks’ centroid. The degradation can be attributed to the lattice damage, non-radiative defects and other defects induced by neutron irradiation.

It is known that the silicon detector is hard to operate above neutron fluence of 1 × 10^14^ n/cm^2^ and the degradation of its CCE is worse than SiC detector after fast-neutron irradiation^12,38^. Hence it can be concluded that the 4H-SiC Schottky diode detectors have a better neutron resistance than silicon detector and could be expected to be well used in fusion neutron detection.
